# Self-Report Measures Assessing Aspects of Personal Recovery in Relatives and Other Informal Carers of Those With Psychosis: A Systematic Review

**DOI:** 10.3389/fpsyg.2022.926981

**Published:** 2022-07-15

**Authors:** Claire Hilton, Steven Jones, Nadia Akers, Katerina Panagaki, William Sellwood

**Affiliations:** ^1^Spectrum Centre for Mental Health Research, Division of Health Research, Faculty of Health and Medicine, Lancaster University, Lancaster, United Kingdom; ^2^Clinical Psychology, Division of Health Research, Faculty of Health and Medicine, Lancaster University, Lancaster, United Kingdom

**Keywords:** caregivers, psychosis, schizophrenia, recovery approach, self-report measures, COSMIN checklist

## Abstract

**Background:**

Providing long-term care for a family member with psychosis can cause significant distress for informal carers due to the trauma of seeing their loved one in crisis, dealing with the difficult symptoms of psychosis and the burden of providing care. An important aspect of carers' adjustment can be construed as their personal recovery in relation to having a relative affected by psychosis. Self-report measures are increasingly used to assess personal recovery in service users, but less is known about the utility of such tools for carers.

**Aims:**

This review aimed to identify all self-report measures assessing aspects of carers' personal recovery, and to quality appraise them.

**Methods:**

Academic Search Ultimate, CINAHL, MEDLINE, PsychINFO and PubMed were searched for articles that reported the development of self-report measures created for carers of those with psychosis. Studies were appraised using the Consensus-based Standards for the Selection of health status Measurement INstruments (COSMIN) checklist. A Levels of Evidence synthesis provided overall quality scores for each measure.

**Results:**

The search identified 3,154 articles for initial screening. From a total of 322 full text articles, 95 self-report measures were identified with a final 10 measures included for the quality assessment showing varying levels of psychometric rigor.

**Conclusions:**

The results show that no single self-report measure is currently available for use to comprehensively assess personal recovery for carers, highlighting the need for further research in this area and the development of a new measure.

## Introduction

Taking on a long-term caring role for a family member who experiences psychosis or schizophrenia is associated with diminished psychological health, grief, social isolation and a poorer quality of life (Awad and Voruganti, 2008; Mulligan et al., 2013; Poon et al., 2017). The prevalence of psychosis is relatively common, with 7% of the adult population experiencing psychosis before their 75th birthday and 50% of these cases occurring before the age of 23 (Mcgrath et al., 2016). The Schizophrenia Commission (2012) have estimated that carers save £1.24 billion of public health funding per year, so it is essential to provide good support to carers. Family carers are also more likely to have financial problems and suffer from interpersonal stress (Mueser and Fox, 2002; Rose et al., 2002). The initial acute phase of treatment for psychosis can be overwhelming and has been compared to a bereavement for the relatives of the service user (Patterson et al., 2005). Carers of those with first episode psychosis have been found to burnt out—feeling exhausted, inadequate, and generally having negative appraisals of their caregiving ability (Onwumere et al., 2018). Carers have described feeling hopeless, depressed, and anxious and this has been conceptualized as a form of secondary trauma that is caused by the ongoing stress of providing long-term care (Wyder and Bland, 2014; Shiraishi and Reilly, 2019). Carers have been found to show symptoms of posttraumatic stress (PTSS) (Hanzawa et al., 2013) such as having intrusive thoughts about the event, feeling alert or on edge a lot of the time, and avoiding difficult thoughts and feelings about their loved ones mental health difficulties. Kingston et al. (2016) found that 44% of carers met the threshold for posttraumatic stress symptoms which was strongly related to negative thinking about themselves, self-blame, and trauma in relation to taking on a caring role. Poon et al. (2017) argue that it is important to acknowledge that families may be struggling with their caring role, and carers often feel isolated and alienated from their usual social support systems (Bland et al., 2009; Hayes et al., 2015). Carers often put their own needs last, but research suggests that when carers attend to their own physical, emotional, and spiritual health that many of their own problems become more manageable (O'Grady and Skinner, 2012). There has been a call for more supportive interventions to be provided for carers (Wyder and Bland, 2014; Poon et al., 2019) both for their own health and wellbeing but also to allow them to provide effective care for the service user (Reine et al., 2003; Testart et al., 2013). For example, recent novel eHealth interventions incorporating psychoeducation and peer support for carers have shown to have a positive impact on carer wellbeing (Lobban et al., 2019; Sin et al., 2019; Batchelor et al., 2022). Taking on a long-term caring role can also alter carers views of self-efficacy and in turn their coping capacity (Wilkinson and Mcandrew, 2008; Rowe, 2012), which may negatively affect both their caring abilities and personal lives (Wyder and Bland, 2014). To better understand and develop more targeted support for carers, it is important to understand their personal experiences (Zendjidjian and Boyer, 2014). Assessing carers experiences is also important in evaluating the treatment and management of care for the service user, as well as evaluating the wellbeing of the carer (Boyer et al., 2016).

An effective method of assessing the experiences of carers is through the use of self-report measures (Richieri et al., 2011) as they are relatively quick to administer and cost effective, which increases the feasibility of incorporating them into routine clinical practice. Self-report measures can also be used to measure the effectiveness of psychosocial and family interventions and can be a useful clinical tool, enabling carers a chance to reflect on their progress over time. The EUFAMI (2014) survey found that assessment of carers experiences was crucial in order to effectively support them, however, despite this need, self-report measures for carers are routinely underutilized in mental health services (Boyer et al., 2016). There are a plethora of measures to assess various aspects of carer experience (Harvey et al., 2005, 2008; Testart et al., 2013) with the majority of measures focusing on the negative aspects of caregiving such as burden, strain, reduced social networks and stigma. There are a few measures that investigate carer coping strategies, perception of need and quality of life (Zendjidjian and Boyer, 2014) and even fewer measures looking at the positive aspects of caring such as, developing greater compassion, finding greater meaning and purpose, and strengthened interpersonal relationships. Understanding the positive aspects of caring has been argued to be an important area to investigate to provide a holistic view of the caring process and to assess what progress is being made (Fulton Picot et al., 1997; Kate et al., 2013; Onwumere et al., 2018). A further important aspect of carer wellbeing that is linked to the positive aspects to caring is the concept of “personal recovery,” conceptualized as living alongside the trauma, burden, stress of caring for a loved one experiencing a psychotic crisis. This is a facet of carers experience that is not assessed by any available measures used for carers but is now widely assessed for service users (Sklar et al., 2013).

The recovery approach has now become a guiding principle in mental health care delivery in most English-speaking countries across the globe (Tew et al., 2012; Slade et al., 2014; Price-Robertson et al., 2017) with the recovery approach being a key UK policy recommendation made by the Department of Health (2011). Personal recovery has been defined as “a deeply personal, unique process of changing one's attitudes, values, feelings, goals, skills and/or roles” and “a way of living a satisfying, hopeful, and contributing life even within the limitations caused by illness” (Anthony, 1993). Personal recovery differs from clinical recovery in that it focuses on the unique personal journey that an individual with a mental health condition goes through in order to find new meaning and purpose in their lives, even in the presence of clinical symptoms (Anthony, 1993; Slade, 2009). There has been very limited research about the recovery approach and carers (Scottish Recovery Network, 2016; Jacob et al., 2017) and recovery informed practice has largely overlooked carers (Hungerford and Richardson, 2013). The bulk of current research has focused on service user recovery, however there is now increasing recognition of “family recovery” (Price-Robertson et al., 2017; Norton and Cuskelly, 2021). Recovery for service users does not happen in isolation and that it is dependent on family support (Wyder and Bland, 2014), and there is a need to understand and support families in their own recovery journey as distinct from the recovery of the service user (Norton and Cuskelly, 2021). It has been argued that carers are on a parallel journey of recovery (Wyder and Bland, 2014; Lovelock, 2016), and that the family recovery journey is intrinsically linked to the service user's journey thus neither can be understood in isolation (Wyder and Bland, 2014). Increasingly there is a call for more recovery focused support for carers and family members (Deane et al., 2015; Estrada, 2016; Poon et al., 2017; Norton and Cuskelly, 2021) and it is seen as important to support the carers recovery journey to assist them in moving forward with their lives by helping them to develop a sense of meaning and purpose despite ongoing challenges (Deane et al., 2015). In supporting carers to identify their own recovery journey, it is also more likely to deepen their understanding of their relatives' experiences of mental health problems by understanding their recovery journey (Lovelock, 2016), which may ultimately lead to improved relationships and a reciprocal support system within the family (Chen and Greenberg, 2004). Supporting the carer's recovery journey may also indirectly support service user's recovery because greater understanding of personal recovery processes gives carers greater confidence in their own “expertise-by-caring” (Fox et al., 2015). There are increasingly more recovery focused family interventions being developed and trialed (Deane et al., 2015; Estrada, 2016; Rue et al., 2016) and there are strong recommendations that carers must be included in recovery oriented social work practice (Poon et al., 2019) and in care planning with mental health professionals (Fox et al., 2015).

In light of the recommendations to provide more recovery-oriented support for carers, there is a requirement to identify self-report measures that may be used to assess personal recovery for carers. However, there are potential challenges in both defining and measuring personal recovery for carers. The primary challenge is that there is a limited literature on what personal recovery may mean for relatives themselves (Wyder and Bland, 2014; Lovelock, 2016). Despite recent systematic reviews of qualitative research examining carers' experiences (Mui et al., 2019; Shiraishi and Reilly, 2019), to date there is no qualitative research exploring specifically what personal recovery means for carers. This presents a potential challenge for this review, as the conceptual understanding of personal recovery will necessarily rely on personal recovery for service users as opposed to their carers. Because of the lack of conceptual literature on personal recovery for carers, there might also be a lack of measures assessing recovery for carers. To the authors' knowledge, there is currently only one measure, that is in the process of development, that focuses on family recovery in particular (Rue et al., 2016; [email] Personal correspondence with K, MacKinnon, 17 August 2016). This has presented a core conceptual problem for this systematic review in that if there is only one specific measure of recovery for carers, is there a need for the review? The authors felt that because of the compelling argument that personal recovery is an important aspect of carer wellbeing then a review looking at measures of various singular dimensions of recovery would reveal which outcome measures could be used together to assess the multi-dimensional nature of personal recovery. Previous systematic reviews looking at carer self-report measures have focused on measures that mainly assess the negative impacts of caring (Harvey et al., 2005, 2008; Testart et al., 2013), with many of the measures reviewed having been developed for the general population. This calls into question the validity of many of the measures in current use because it is difficult to adequately assess the experience of carers from the general population (Hilton, 2016). It is generally accepted to be good practice for self-report measures to be developed using the perceptions of the population they evaluate, to improve the relevance and validity of the measure (Slevin et al., 1988; Testart et al., 2013). In addition, previous reviews (Harvey et al., 2005, 2008; Testart et al., 2013) found a limited amount of self-report measures related to positive outcomes, such as quality of life, however, none of the reviews identified a measure that related to the concept of recovery. Therefore, there is a need for a more up to date review that focuses on aspects related to the recovery concept, and where the self-report measures reviewed have been developed specifically for the carer population.

The primary aim of this review was to identify all self-report measures that have been developed for use with carers of those with psychosis or schizophrenia, and that assess aspects of personal recovery. A quality appraisal of the psychometric properties of the self-report measures was carried out using the COSMIN checklist (Mokkink et al., 2010). This review had two further aims: to investigate and assess the level of carer involvement in the development of each self-report measure, and to explore how well personal recovery was assessed by each self-report measure.

## Methods

### Protocol and Registration

This systematic review was registered on 22nd May 2018 with PROSPERO (CRD42018096020), and followed the PRISMA (Moher et al., 2009) guidelines.

### Eligibility Criteria

Quantitative and mixed method studies that used a self-report measure(s) to assess the health and wellbeing of carers of those with psychosis or schizophrenia, were included. Carers included: parents, spouses, partners, grandparents, siblings, adult children, extended family and close friends in a caring role. Studies assessing paid carers, in-patient care staff and relatives under the age of 18 (young cares) were excluded. It was thought likely that adults and adolescents/children would have substantially different experiences because of varying levels of responsibility and role expectations. The clinical group of interest were service users who had received a diagnosis of psychosis (acute, chronic, first episode) or schizophrenia (all types). Service users who have experienced an episode of psychosis as part of another serious mental illness such as bipolar disorder or personality disorder were also included in this review, but only if the psychotic episode was the main focus of the article. See Appendix A for a full list of inclusion and exclusion criteria.

The self-report measures included any formally tested measure such as questionnaires, surveys, outcome assessments, instruments, and rating scales. Only self-report measures developed and validated in the English language and designed specifically to assess carers of those with a mental health diagnosis were included. There was no limitation on the date range of publication. Modified and brief versions of self-report measures were excluded from this review.

The conceptual challenge of this review has been the fact that there is limited research on personal recovery for carers, so particular attention was paid to operationalize this concept. Since there are no available self-report measures that primarily assess personal recovery for carers, several linguistic terms of recovery were collated from key authors on the topic of personal recovery (Anthony, 1993; Resnick et al., 2005; Slade, 2009; Leamy et al., 2011). These linguistic terms were discussed by the research team and a checklist of terms was created and incorporated as part of the search strategy for this review (see Supplementary Material for a copy of the checklist).

### Information Sources

The following databases were searched in September 2017 with an updated search in March 2022: Academic Search Ultimate, CINAHL, MEDLINE, PsychINFO and PubMed. Additional searching strategies included checking the reference lists and citation tracking (using Web of Science) of the final papers. The search strategy involved setting out three distinct categories related to the key elements of the review: population, type of instrument and construct. Database specific search strategies were developed utilizing tools such as MESH headings (MEDLINE) and thesaurus terms (PsychINFO). See Appendix B for an example search strategy.

The following key word search terms were used to search all databases: [POPULATION] carer^*^, caregiver^*^, relative^*^, families, family caregiver^*^, psychosis, psychoses, psychotic, psychotic disorder, schizophren^*^, [TYPE OF INSTRUMENT] outcome measure, instrument^*^, assessment, measurement scale, rating scale, survey, questionnaire, patient reported outcome measure, self-report measure, [CONSTRUCT] recovery, mental health recovery, hope, optimism, goals, relationships, identity, meaning, personal responsibility, full engagement with life, empowerment, knowledge, life satisfaction, self-direction, full potential, person-driven, peer support, support groups, community, strengths, respect, motivation to change, positive thinking, valuing success, aspirations, positive sense of identity, quality of life, meaningful life, meaningful social roles, rebuilding life, employment, self-efficacy, coping, and adaptability.

### Quality Appraisal

The COSMIN checklist (Mokkink et al., 2010) was used for this review as the gold standard for providing a comprehensive assessment of the psychometric properties of self-report measures (Rosenkoetter and Tate, 2018). The COSMIN checklist was developed by expert consensus (Mokkink et al., 2010), is freely available and includes a thorough user manual and scoring sheet and as such provides a consistent and transparent approach to systematic reviews of self-report measures.

### Data Extraction

Online data extraction forms were created on DistillerSR (Evidence Partners, 2011) for the title and abstract screening and full text screening. Two independent reviewers (CH and NA) assessed all the title and abstracts against the inclusion criteria. Separate scoring sheets were used for the COSMIN 4-point checklist results, and for the assessment of quality of measurement properties per measure. CH carried out the COSMIN assessment, and then NA carried out a 20% check of the COSMIN results. Data were extracted by CH from the final 15 measure development or validation papers that related to: (1) details about the measures (2) characteristics of the study participants (3) details about the development of the measure and the psychometric properties required for the COSMIN assessment.

### Synthesis of Results

The results of the COSMIN checklist were synthesized into two main results tables. The first table summarized the methodological quality of each study per measurement property (**Table 3**). Due to the comprehensive nature of the psychometric properties assessed, the COSMIN checklist does not provide one single overall score for each measure. Therefore, a second table (**Table 4**) was created to provide an overall assessment of the measurement properties for each outcome measure. The main psychometric properties assessed by the COSMIN checklist are: internal consistency, reliability (test re-test), content validity, structural validity and hypothesis testing. Certain psychometric properties assessed using the COSMIN checklist, such as cross-cultural validity, were not included in this review as no data were reported in the measure development papers.

## Results

### Study Selection

**Figure 1:**
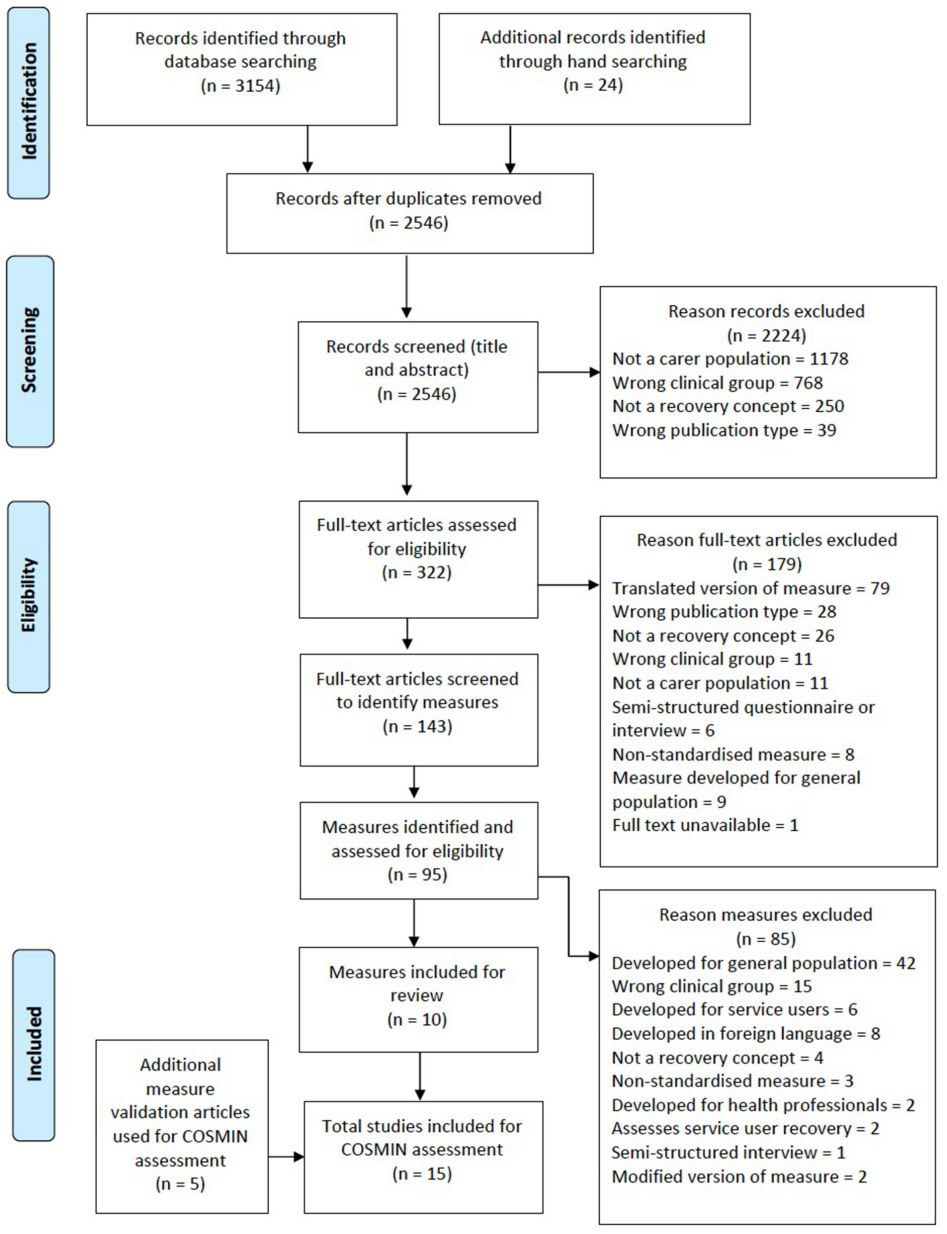
Flow chart detailing the literature search.

The electronic database search identified 3,154 records with an additional 24 records identified through other search methods. The title and abstracts were screened by two reviewers independently (CH and NA) with good inter-rater reliability (Cohen's κ = 0.78). A total of 322 full text articles were selected based on the title and abstract screening. Of the 322 full text articles, 179 were excluded because they were based on a translated version of a measure, did not assess the psychometric properties of a measure or did not assess an aspect of recovery. This resulted in a total of 143 full text articles being screened to identify any potentially relevant outcome measures, of which 95 self-report measures were identified. Only 15 studies, covering ten measures, fulfilled the inclusion criteria. The main reasons for exclusion at full text stage are presente in Figure 1.

Table 1 shows the characteristics of included measures, Table 2 shows the characteristics of the included studies, and Table 3 details the COSMIN review carried out on the included studies to assess their methodological quality. No study was excluded based on methodological quality. A synthesis of the COSMIN results of all studies is summarized in a levels of evidence table (Table 4) where an assessment of all the measurement properties was carried out per measure. Supplementary Material details the quality criteria used to assess the levels of evidence for each measure in Table 4 and is based on Terwee et al. (2007) and de Vet et al. (2011) (see Appendix C).

**Table 1:** Characteristics of included measures.

**Instrument**	**Authors**	**Target** **population**	**Country** **of origin**	**Year of** **development**	**Constructs** **assessed**	**Domains**	**Number of** **subscales** **(number of** **items)**	**Response options**	**Full copy of** **instrument** **available**
Carer Coping Style Questionnaire (CCSQ)	Budd et al.	Carers of those with schizophrenia	UK	1998	Coping styles	Copying style subscales— collusion; reassurance; emotional over-involvement; constructive; resignation; passive; warmth; criticism/coercion; over-protectiveness	9 (89)	5-point Likert scale	Yes
Carer Well-being and Support Questionnaire (CWS)	Quirk et al.	Carers of those with serious mental illness and dementia	UK	2009 and 2012	Wellbeing and support	Subscale 1—Carer wellbeing scale (10 domains): your day-to-day life; your relationship with the person you care for; your relationships with family and friends; your financial situation; your physical health; your emotional wellbeing; stigma and discrimination; your own safety; the safety of the person you care for; your role as a carer. Subscale 2 - Carer support (5 domains): information and advice for carers; your involvement in treatment and care planning; support from medical and/or care staff; support from other carers; and taking a break (respite).	2 (49)	4 and 5-point Likert scales	Yes
Care-related Quality of Life (CarerQol)	Brouwer et al.	Carers of those with serious mental and physical illness	Netherlands	2006	Quality of life	7 dimensions exploring burden: fulfillment, relational, mental health, social, financial, support, physical, and 1 dimension exploring happiness	2 (8)	Mixed format: single choice answers and a VAS	Yes
Carers' and users' expectations of services—carer version (CUES-C)	Lelliott et al.	Carers of those with serious mental illness	UK	2003	Experiences of caregiving	13 dimensions: help and advice, information about care workers, information about mental illness, involvement and planning of care, support for carers, own life, relationships, family and friends, money, wellbeing, stigma and discrimination, risk and safety, choice to care.	13 (26)	Normative statements with a 3-point rating scale, free-text response section	Carer Wellbeing and Support Questionnaire (CWS) replaced this.
Experience of Caregiving Inventory (ECI)	Szmukler et al.	Carers of those with serious mental illness	UK and Australia	1996	Experience of caregiving	8 negative (difficult behaviors; negative symptoms; stigma; problems with services; effects on family; the need to provide backup; dependency; loss), 2 positive (rewarding personal experiences; good aspects of the relationship with the patient)	10 (66)	5-point Likert scale	Yes
Family Mental Health Recovery Evaluation Tool	Rue et al.	Families of those with serious mental illness.	USA	2016	Positive aspects of caregiving, family recovery	Capacity to Support Family Member, Hopefulness toward Recovery, Mental Health Coping Skills, Boundaries and Role Clarification, Communication, Self-Efficacy toward Recovery	6 (46)	Mixture of 3 and 5-point Likert scales	No
Friedrich-Lively Instrument to Assess the Impact of Schizophrenia on Siblings (FLIISS)	Friedrich et al.	Siblings of those with schizophrenia	USA	2002	Stress and caregiving	Primary stressors (caregiving roles, reactions to caregiving, disturbing behaviors, homelessness, alcohol, drugs, relationship with ill sibling). Secondary stressors (relationships with parents and family, relationship with other siblings, concerns about own children, relationship with spouse, relationship with friends, school performance, work performance and career). Mediators of stress: coping strategies (emotional/spiritual, relationships, cognitive and action) and social support (from friends, relatives, professionals and organized groups). Outcomes (effect on health, view of self)	5 (256)	Mixture of Likert scales, multiple choice answers and specific answers	Yes
North-Sachar Family Life Questionnaire (N-SFLQ)	North et al.	Carers of those with schizophrenia	USA	1998	Experience of caregiving	Coping strategies, knowledge of illness, communication, behavior management, employment	5 (11)	5-point Likert scale	Yes
Schizophrenia Caregiver Questionnaire (SCQ)	Gater et al.	Carers of those with schizophrenia	USA, and with an international validation	2015 and 2016	Experiences of caregiving	Two distinct constructs: “Humanistic impact”—social, emotional, daily life and physical impact; “Aspects related to caregiver role”—perceptions of caregiving, financial impact.	13 (30)	11-point numerical rating scale (NRS)	Yes
Social Network Questionnaire (SNQ)	Magliano et al.	Carers of those with schizophrenia	Across Europe	1998	Social networks	Quality and frequency of social contacts, practical social support, emotional support, the presence and quality of an intimate supportive relationship.	4 (15)	Not reported	Yes

**Table 2:** Characteristics of included studies.

**Study**	**Population**	**Sample** **size**	**Age, mean (SD** **or range)**	**Female (%)**	**Country**
*CCSQ* Budd et al. (1998)	Carers of those with schizophrenia	91	59 (20–85)	71	UK
*CWS* Quirk et al. (2012)	Carers for those with mental health problems and dementia	361	65.5 (13.1)	65.3	UK
*CarerQol* Brouwer et al. (2006)	Carers of those with physical and mental health problems	175	60.8 (13.1)	75	Netherlands
Hoefman et al. (2011)	Carers of those with physical and mental health problems	275	58.74 (12.74)	74.3	Netherlands
Hoefman et al. (2013)	Carers of those with physical and mental health problems	1,244	<47.1–47.1%	58.3	Netherlands
*CUES-C* Lelliott et al. (2003)	Carers of those with mental health problems	243	60 (24–87)	Approx. 75	UK
*ECI* Joyce et al. (2000)	Cares for those with psychosis	69	Not reported	Not reported	UK
Szmukler et al. (1996)	Carers of those with mental health problems	626	1st sample−53 (+−30 years), 2nd sample - 46 (+−15 years)	66 (1st and 2nd samples combined)	UK and Australia
*Family Mental Health Recovery Evaluation Tool* Rue et al. (2016)	Carers of those with mental health problems	108	<40–86%	89.9	USA
*FLIISS* Friedrich et al. (2002) (Part 1 paper)	Siblings of those with schizophrenia	N/A^*^	N/A^*^	N/A^*^	USA
Rubenstein et al. (2002) (Part 2 paper)	Siblings of those with schizophrenia	761	39.7 (10.6)	73.7	USA
*N-SFLQ* North et al. (1998)	Carers of those with schizophrenia	56	Not reported	53	USA
*SCQ* Gater et al. (2015)	Carers of those with schizophrenia	19	51.63 (28–69)	79	USA
Rofail et al. (2016)	Carers of those with schizophrenia	358	Not reported	Not reported	Argentina, Brazil, Canada, Germany, Spain, France, UK, Italy
*SNQ* Magliano et al. (1998)	Carers of those with schizophrenia	236	Not reported	Not reported	UK, Greece, Italy, Portugal and Germany

**Table 3:** COSMIN results showing the methodological quality of each study per measurement property.

**Name of measure and study**	**Internal consistency**	**Reliability**	**Content validity**	**Structural validity**	**Hypothesis testing**
*CCSQ*					
Budd et al. (1998)	Poor	–	Poor	Poor	Fair
*CWS*					
Quirk et al. (2012)	Excellent	Fair	Excellent	Excellent	Good
*CarerQol*					
Brouwer et al. (2006)	-	-	Excellent	-	Fair
Hoefman et al. (2011)	-	-	Fair	-	Fair
Hoefman et al. (2013)	-	-	Excellent	-	Fair
*CUES-C*					
Lelliott et al. (2003)	-	Fair	Good	Fair	-
*ECI*					
Szmukler et al. (1996)	Excellent	-	Excellent	Excellent	Good
Joyce et al. (2000)	-	-	-	-	Fair
*Family Mental Health Recovery Evaluation Tool*					
Rue et al. (2016)	Poor	-	Fair	Poor	-
*FLIISS*					
Friedrich et al. (2002) (Part 1 paper)	-	-	Excellent	-	-
Rubenstein et al. (2002) (Part 2 paper)	Poor	-	-	Poor	Good
*N-SFLQ*					
North et al. (1998)	-	-	-	-	-
*SCQ*					
Gater et al. (2015)	-	-	Excellent	-	-
Rofail et al. (2016)	Excellent	Good	-	Excellent	Fair
*SNQ*					
Magliano et al. (1998)	Poor	Fair	Fair	Fair	-

**Table 4:** Quality of measurement properties per self-report measure.

**Outcome measure**	**Internal consistency**	**Reliability**	**Content validity**	**Structural validity**	**Construct validity (Hypothesis testing)**
CCSQ	+	N/A	-	-	+
CWS	-	+	+	+	+
CarerQol	N/A	N/A	-	N/A	+
CUES-C	N/A	-	+	-	N/A
ECI	+	N/A	+	+	+
Family mental health recovery evaluation tool	+	N/A	-	?	N/A
FLIISS	-	N/A	+	?	+
N-SFLQ	N/A	N/A	N/A	N/A	N/A
SCQ	+	+	+	?	+
SNQ	-	-	+	+	N/A

### Results of Individual Studies

Presented below are the summary findings of each measure, listed in alphabetical order by title of the measure. Each summary provides an overview of the constructs assessed by the measure, whether the constructs are based on theoretical model(s) and a summary of the theoretical model(s) used, the overall structure of the measure (domains and sub-scales), the response options, an assessment of the psychometric quality of the measure based on the COSMIN checklist, the level of public involvement in the development of the measure, and finally how the measure relates to the concept of personal recovery. All outcome measures assessed in this review have been specifically created for use with carers of those with psychosis and schizophrenia.

#### Carer Coping Style Questionnaire (CCSQ)

The Carer Coping Style Questionnaire (CCSQ; Budd et al., 1998) was designed to assess the coping styles of carers of those with schizophrenia and was based on two theoretical models; assessing the four dimensions of expressed emotion (Leff and Vaughan, 1985), and the seven coping styles identified by Birchwood and Cochrane (1990). The CCSQ has 89 items divided into nine subscales (collusion, reassurance, emotional over-involvement, constructive, resignation, passive, warmth, criticism/coercion and over-protectiveness). The response format of the CCSQ is a 5-point Likert scale. The CCSQ was tested on 91 carers of those with schizophrenia in the United Kingdom. It scored “poor” for internal consistency on the COSMIN checklist because the authors did not conduct a factor analysis or principal components analysis on the results despite a good alpha score for each subscale (Cronbach's alpha ranged between 0.69 and 0.87). Even if the authors had carried out a factor analysis, according to the COSMIN criteria, the CCSQ has a poor sample size (*n* = 91) for testing the unidimentionality of the factors as the population was below five times the number of items on the scale (89 items). The CCSQ scored “poor” on content validity because they did not involve carers in the development of the measure, meaning it is not possible to say that the items were relevant to the study population. The authors generated an item pool based on the theoretical models and then carried out a Q-sort with a team of health professionals to classify the items into discrete categories with the final item similarity matrix being subjected to a cluster analysis. Because no principal components analysis or factor analysis was carried out the CCSQ scored “poor” on structural validity. The CCSQ demonstrates “fair” hypothesis testing as the authors did not make it explicit how missing items were handled and it was unclear what a priori hypotheses were made. The CCSQ showed concurrent validity compared to the General Health Questionnaire (GHQ-28) (Goldberg, 1978), the Cost of Care Scale (CCS) (Kosberg and Cairl, 1992), and the Symptom-Related Behavioral Disturbance Scale (SBDS) (Birchwood, 1983).

The CCSQ does not seem to assess many aspects related to carer's personal recovery as the items assess carer coping styles in relation to their interactions with the service user and how this relates to expressed emotion. The CCSQ does not focus on the personal experiences of the carers, rather their interactions with the service user and because of this the CCSQ does not seem to fit well with the recovery framework.

#### Carer Wellbeing and Support Questionnaire (CWS)

The CWS (Quirk et al., 2009) assesses the well-being and support of carers of those with serious mental illness and dementia and was based on a pre-existing measure called the Carers' and users' expectations of services—carers' version (CUES-C) (Lelliott et al., 2003). The CWS consists of 49 items and is divided into two subscales: the carer well-being scale with 10 domains (your day-to-day life; your relationship with the person you care for; your relationships with family and friends; your financial situation; your physical health; your emotional wellbeing; stigma and discrimination; your own safety; the safety of the person you care for; your role as a carer), and the carer support scale with 5 domains (information and advice for carers; your involvement in treatment and care planning; support from medical and/or care staff; support from other carers; and taking a break (respite). The CWS sub-scales are scored using either a 4 or 5-point Likert scale depending on the specific subscale. The CWS was also validated with a large population sample of 361 carers from various centers across the United Kingdom. The CWS scored “excellent” on the COSMIN checklist for internal consistency as they reported high Cronbach's alpha scores for each subscale (0.96 and 0.97, respectively). The CWS scored “fair” for reliability on the COSMIN checklist only because the authors did not state the time interval between the two administrations of the test. The intra-class correlations for both subscales were high: *r* = 0.92 (*n* = 91) for the carer wellbeing scale and *r* = 0.88 (*n* = 92) for the carer support scale which demonstrates good test-retest reliability. The CWS showed “excellent” content validity as the measure went through a rigorous three phase construction process to make sure items were relevant to the constructs being assessed, and relevant for the target population. Carers were consulted regularly throughout the development and validation stages of the CWS construction which demonstrates excellent face validity and follows current good practice guidelines for questionnaire construction (Streiner et al., 2015). The CWS demonstrated “excellent” structural validity as the two-factor model accounted for over 50.8% of the variance. The CWS also showed “good” construct validity with all convergent hypotheses supported by moderately high correlations with the General Health Questionnaire (GHQ-12) (Goldberg, 1978) (*r* = −0.66, *n* = 194) and the Involvement evaluation questionnaire – European version (IEQ-EU) (Van Wijngaarden, 2003) (*r* = −0.70, *n* = 122).

The CWS covers a broad range of issues for carers and fits well with the recovery framework. The first sub-scale (Carer Wellbeing) is particularly relevant to the recovery framework as it covers carers personal experiences and looks at the various aspects of wellbeing such as physical health, mental health, financial resources, social networks, the carers own needs and how the carers view the future. The second sub-scale (Carer Support) is more focused on the level and quality of support that carers receive from mental health services and is not as directly relevant to the recovery framework as it focuses more on the practical aspects of caring and not how the carer perceives or finds meaning in their role. The authors do suggest that the CWS can be used as in mix-and-match combinations and that the validated wellbeing and support subscales can be administered separately, which could mean that just the wellbeing sub-scale could be used to measure those aspects of recovery.

#### Care-Related Quality of Life (CarerQol)

The CarerQol (Brouwer et al., 2006) was developed to measure the quality of life of carers of those with physical and mental health problems. Eight items are divided into two subscales, with seven items relating to burden (fulfillment, relational, mental health, social, financial, support, physical) and one item to assess happiness. The response format is mixed, with single choice answers for the burden subscale, and a visual analog scale (VAS) for the happiness item. The CarerQol has been well-validated for content and construct validity with three validation studies (Brouwer et al., 2006; Hoefman et al., 2011, 2013) all based on data from carer populations in the Netherlands. It is unclear as to whether the data were collected using the English or Dutch version of the CarerQol, however, it was decided to include this measure in the review as the measure is available online in the English language. All three studies had large sample sizes (Brouwer et al., 2006, *n* = 175; Hoefman et al., 2011, *n* = 1244; Hoefman et al., 2013, *n* = 275). Based on the COSMIN criteria two out of the three studies scored “excellent” for content validity (Brouwer et al., 2006; Hoefman et al., 2013). The CarerQol scored less well for hypothesis testing with all three studies scoring “fair,” the main reason being that the studies either failed to provide a description of how the missing items were handled or they failed to report on whether any a priori hypotheses were formulated. Even though three validation studies were carried out, there was no assessment of the measure's internal consistency, reliability or structural validity. The CarerQol did show some level of carer input in the development of the measure which is positive in terms of participant involvement. Carers were involved in some initial pilot testing and in commenting on the wording of the items, however, the researchers were solely responsible for devising the initial item pool.

The CarerQol does not fit well within the recovery framework despite purporting to assess carer quality of life. The bulk of the items relate to aspects of carer burden with only one item relating to happiness.

#### Carers' and Users' Expectations of Services—Carer Version (CUES-C)

The CUES-C (Lelliott et al., 2003) assesses the experience of caregiving based around 13 items (help and advice, information about care workers, information about mental illness, involvement and planning of care, support for carers, own life, relationships, family and friends, money, wellbeing, stigma and discrimination, risk and safety, choice to care). The response format involves three questions per item (which is worded as a normative statement). Part A questions ask whether the carers experiences matches the items normative statement, part B questions ask if the carer would like further support in that area, part C is a free text box for comments on that item. It was developed for use with carers of those with mental health problems in the United Kingdom. It is worth noting that this measure was deconstructed and used as the basis for the development of the CWS. The CUES-C was validated with a good size sample of 243 participants; however, it did not score well on the COSMIN checklist. The CUES-C scored “fair” for reliability on the COSMIN checklist because the authors did not report on how missing items were handled. Interclass coefficients were calculated for test-re-test reliability and were moderately good for both parts of the measure (*r* = 0.61, *n* = 97). The CUES-C was not based on any kind of theoretical model and as such it would be difficult to assess if all items together adequately reflect the construct being measured, which relates to content validity. Despite of this, the CUES-C scored “good” for content validity because they showed a very good level of carer involvement at all stages of the questionnaire development. An advisory panel worked with the authors throughout the development process providing feedback on the measure and the authors conducted focus groups and individual interviews on the draft measure. The CUES-C scored “fair” for structural validity on the COSMIN checklist because there was no description of how missing items were handled. The authors did carry out a comprehensive principal components analysis on both parts of the measure, part A includes 3 factors that account for 49% of the variance and part B includes 2 factors that account for 51% of the variance.

The CUES-C has several items that fit with the recovery framework, such as the statements about the carer's own lives, relationships with the service user, relationships with family and friends, their own wellbeing that includes both positive and negative elements, and their personal choice to care.

#### Experience of Caregiving Inventory (ECI)

The ECI (Szmukler et al., 1996) was the most commonly used measure in this review, being used in 20 of the 95 studies reviewed. The ECI provides a very broad view of the experiences of caregiving and is based on the stress-appraisal-coping framework (Lazarus and Folkman, 1984). It assesses both negative and positive aspects of caring with 66 items divided across 10 domains. There are eight domains covering the negative aspects of caring (difficult behaviors, negative symptoms, stigma, problems with services, effects on family, the need to provide backup, dependency, and loss), and two domains covering the positive aspects of caring (rewarding personal experiences, and good aspects of the relationship with the patient). The response format for the ECI is a 5-point Likert scale and it was developed by a team of researchers in the United Kingdom and Australia. The ECI has been validated by two studies, the original by Szmukler et al. (1996) that provided a good overall assessment of most of the psychometric properties of the measure, and a subsequent study by Joyce et al. (2000) that assessed hypothesis testing. On the COSMIN checklist, the ECI showed “excellent” internal consistency (Szmukler et al., 1996) as it had a large sample size (*n* = 626) and good Cronbach's alpha scores that were calculated for each dimension (ranging from 0.74 to 0.91). The ECI also demonstrates “excellent” content validity as it went through a rigorous five stage development process where carers had a high level of input at every stage of its development. For example, items were devised through a series of one-to-one interviews and focus groups with 120 carers. Szmukler et al. (1996) also ensured that the items were validated within the stress-coping model and found that the ECI predicted psychological morbidity. The ECI also scored “excellent” for structural validity because the authors carried out a comprehensive principal components analysis on a large sample of 626 carers. The initial 14 factor model accounted for 60% of the variance, and this was refined down to 10 factors for the final measure. The ECI scored “good” on the Szmukler et al. (1996) study and “fair” on the Joyce et al. (2000) study for hypothesis testing. This was because they did not state the expected magnitude of correlations or differences in the Szmukler et al. (1996) paper, and because only limited information was provided on the measurement properties of the comparator instruments in the Joyce et al. (2000) paper.

The ECI partially fits with the recovery framework because there are two dimensions that focus on the positive aspects of caring: “positive personal experiences” that assesses learning about oneself, having greater confidence, and being more understanding of others with problems; and “good aspects of the relationship” that assesses the relationship with the service user and whether the carer feels a sense of self efficacy in their care provision. However, a large portion of the ECI looks more at the burden of caring, such as stigma, dependency, and loss, and dealing with difficult behaviors and negative symptoms, which does not fit with the recovery framework.

The Brief Experience of Caregiving Inventory (BECI) (O'Driscoll et al., 2018) provides a shortened 19-item version of the ECI, which aims to provide a quicker and less burdensome version for carers to complete. The BECI was reviewed but excluded from the final COSMIN assessment for two reasons. First, the BECI has not been validated using a new sample population, as the authors carried out a Multidimensional Item Response Theory (MIRT) on the original data collected for the validation of the ECI in 1996. It is not possible to carry out a COSMIN assessment without a full validation paper with data collected from a relevant sample population. Secondly, part of the exclusion criteria for this review was to exclude modified versions of self-report measures.

#### Family Mental Health Recovery Evaluation Tool (Provisional Title)

The Family Mental Health Recovery Evaluation Tool (FMHRET; Rue et al., 2016) was developed to assess the wellbeing and recovery of family members who were taking part in an online family recovery intervention (Families Healing Together, 2018) in the USA and was validated by Rue et al. (2016). The intervention is based on the stress-appraisal-coping framework (Lazarus and Folkman, 1984) and the constructs assessed are the positive aspects of caregiving and family recovery. The measure contains 46 items divided into six domains (capacity to support family member, hopefulness toward recovery, mental health coping skills, boundaries and role clarification, communication, self-efficacy toward recovery). The response options are divided into a mixture of 3 and 5-point Likert scales. The FMHRET did not score well overall on the COSMIN checklist mainly because of the small sample size used to validate the measure. The authors used a sample of 108 carers, which is less than five times the number of items on the measure. To score anything above “poor” on the checklist, the measure should have had a sample size of more than 230 carers. The FMHRET scored “poor” for internal consistency but did demonstrate strong alpha values (α = 0.76–0.86). It scored “poor” for its structural validation because of the small sample size. It should be noted that the authors only intended to carry out an exploratory factor analysis for this study, which may have been one of the reasons for the small sample size. The exploratory factor analysis of the FMHRET showed a five-factor model that accounted for 47% of the variance. The FMHRET scored “fair” for content validity, again because of the small sample size and because they didn't employ robust participant involvement in the development of the measure. According to the authors, the initial items were developed through a qualitative analysis of blog post entries from the “Families Healing Together” intervention, with a subsequent construct validity assessment with five “experts” to refine the conceptual definitions. It is not made clear who the “experts” were but following communication with one of the authors, it was clarified that only one of the “experts” was a carer (K. MacKinnon, personal communication, August 19, 2016).”

Of all the measures assessed in this review, the FMHRET is the most well-positioned within the recovery framework because it was developed to assess family recovery specifically. It looks at the positive aspects of caring as its primary construct but also includes other aspects such as coping skills and self-efficacy. Unfortunately, at the time of writing this review, the measure was not available for use outside of the “Families Healing Together” intervention.

#### Friedrich-Lively Instrument to Assess the Impact of Schizophrenia on Siblings (FLLISS)

The FLLISS (Friedrich et al., 2002) measures the stress of caregiving for siblings of those with schizophrenia and is based on the stress model of caregiving (Pearlin et al., 1990). The FLLISS was developed in the USA. It consists of 256 items across five domains that cover primary stressors, such as: caregiving roles, disturbing behaviors and their relationship to the ill sibling; secondary stressors such as: relationships with friends and family, work performance and career; the mediators of stress such as: coping strategies and social support; and outcomes such as: effect on health and view of self; and some demographic questions. The FLLISS uses a mixture of Likert scales, multiple and single choice answers. The FLLISS was validated in two parts, the first part reporting how the measure was devised (Friedrich et al., 2002) and the second part reporting the validation of the psychometric properties of the FLLISS (Rubenstein et al., 2002). The FLLISS scored “excellent” on the COSMIN checklist for content validity as the authors had a very rigorous approach in the development of the measure, basing the content of the items on a qualitative content analysis of interview data from 30 siblings. The authors also used some of the direct wording from the interview statements in the wording of the items which the authors claim increased the ecological validity and relevance of the measure for siblings, unfortunately they do not indicate which items are based on the interview statements in their published article. Siblings were also invited to comment on the final version of the measure before testing. The FLLISS scored “poor” for internal consistency because the sample size used was less than five times the number of items on the measure despite having a large sample of 761 participants. The FLLISS is the longest measure in this review with 256 items and the study would have needed a sample of over 1,280 to score over a “poor” rating on the COSMIN checklist. This sample size issue also affected the score for the structural validity of the FLLISS, which was also “poor” while all the rest of the scores were “good” to “excellent.”

Even though the FLLISS is mainly concerned with assessing primary and secondary stressors, there are still elements to the measure that fit well with the recovery framework. Within those domains are items that assess the relationships between siblings, their family and friends, and topics like career and employment. Also, the FLLISS has a section that looks at the mediators of stress which is more relevant to the recovery framework as this assesses coping strategies and social support. The one concern in considering this measure for use to assess recovery is that it was specifically designed and validated for siblings of those with schizophrenia and as such it's unclear as to whether it could be used with other family carers.

#### North-Sachar Family Life Questionnaire (N-SFLQ)

The N-SFLQ (North et al., 1998) assesses the experience of caring for someone with schizophrenia and was not based on any sort of theoretical framework. It consists of 11 items set across five domains that cover: coping strategies, knowledge of the illness, communication, behavior management, and employment. It is rated on a 5-point Likert scale. The N-SFLQ was designed for and piloted in a small pilot study (*n* = 56) of a family intervention training program in the USA. No formal validation was carried out for this measure, which rendered it impossible to assess its psychometric properties using the COSMIN checklist.

This measure covers some of the aspects related to the recovery framework, such as coping strategies, communication and employment, however, it appears that there is also a large focus on the service user and their progress with items assessing number of hospital admissions and length of hospital stay. Additionally, this measure has no formal validation and because of these reasons, it is not recommended for use in assessing recovery in carers.

#### Schizophrenia Caregiving Questionnaire (SCQ)

The SCQ (Gater et al., 2015) was specifically designed for carers of those with schizophrenia and assesses their experiences of caregiving. It was not based on any theoretical framework but was developed from a commonly used burden measure called the Zarit Burden Interview (ZBI) (Zarit et al., 1980). The SCQ has 30 items spread across 13 domains grouped into two main constructs of the “humanistic impact” of caring, and “aspects related to the caregiver role.” The response format is an 11-point numerical rating scale. The SCQ was validated in two parts. The first validation paper by Gater et al. (2015) assessed the content validity of the measure and outlined how the measure was devised. On the COSMIN checklist, the measure scored “excellent” for content validity. The authors describe a high level of participant involvement in the development of the measure as they carried out in-depth qualitative interviews with 19 carers to discuss the measure using a cognitive debriefing technique to assess their understanding of the measure and whether it was relevant and comprehensive for carers. The authors claim the measure demonstrates strong face validity. The second validation for the SCQ (Rofail et al., 2016) assessed the psychometric properties of the measure. The SCQ scored “excellent” for internal consistency with Cronbach alpha scores ranging between 0.80 and 0.96. Rofail et al. (2016) also assessed the test-retest reliability (*r* = 0.75−0.87) demonstrating “good” reliability on the COSMIN checklist. The SCQ showed “excellent” structural validity with a comprehensive factor analysis where 13 clear domains were identified. The SCQ scored “fair” for hypothesis testing. Even though the authors report that the item domain validity was fully satisfactory and that it showed good item convergent and divergent validity, according to the COSMIN checklist the SCQ scored “fair” because it was not made apparent what the a priori hypotheses were regarding the correlations or mean differences were.

In terms of the recovery framework, the SCQ seems to have a good fit. Even though it is based on a burden interview (ZBI) the domains assessed seem directly relevant to aspects of the recovery approach. For example, the SCQ assesses the “humanistic impact” of caring relating to the social, emotional, physical impacts on the carer's daily life, while the “aspects related to the caring role” investigates the carers perceptions of caregiving and the financial impact. It is a very well-validated measure with excellent participant involvement throughout the development process and as such would be a strong measure to use to assess aspects of carer recovery.

#### Social Network Questionnaire (SNQ)

The SNQ (Magliano et al., 1998) was designed to assess social networks and was developed for use with carers of those with schizophrenia. The measure was not based on any kind of theoretical framework but was based on the wider literature on social networks (L. Magliano, personal communication, August 2, 2016). The SNQ contains 15 items with four domains assessing the quality and frequency of social contacts, practical social support, emotional support, and the presence and quality of an intimate supportive relationship. The validation of the SNQ was discussed within a paper that reports the results of a large European research trial (Magliano et al., 1998) and as such there is limited detail about how the measure was developed. The SNQ scored “fair” for internal consistency on the COSMIN checklist primarily because the authors did not describe how missing items were handled. The SNQ had moderate Cronbach's alpha values ranging between 0.56 and 0.75 for each of the four factors. The test re-test of the SNQ was carried out with 50 carers 10 days apart however the SNQ scored only “fair” on the COSMIN checklist for reliability because it was not explained how missing items were handled. The SNQ scored “fair” for content validity as the authors did not describe whether they assessed all items as being relevant to the construct being measured and did not base the measure on a theoretical framework. There did not appear to be much participant involvement in the development of the measure apart from carers providing comments on the comprehensibility and relevance of the items on a trial version of the SNQ. To assess the structural validity of the SNQ the authors carried out a factor analysis and found four distinct factors that accounted for 56% of the variance, however, SNQ scored “fair” for structural validity as it was not clear how missing items were handled.

The SNQ is the only measure to provide a comprehensive assessment of social networks which fits well with this aspect of the recovery framework; however, this is only a part of the recovery journey that carers may travel. For example, it does not cover whether carers have developed a greater sense of meaning and purpose through caring, or whether they feel more confident and empowered to rebuild their lives. Because of the this the SNQ should not be used in isolation to assess recovery but could be used in conjunction with other measures to create a suite of questionnaires to comprehensively assess recovery for carers.

### Additional Analysis

The overall findings from the COSMIN assessment of all 15 studies was synthesized into a levels of evidence table (Table 4) following the approach outlined in de Vet et al. (2011). This provides a good overall summary of the quality of each psychometric property for each of the 10 outcome measures reviewed. The quality criteria for each psychometric property used for this assessment were based on the recommendations by Terwee et al. (2007) and is outlined in Appendix C.

## Discussion

### Summary of Evidence

The aim of this review was to identify self-report measures created for carers of those who experience psychosis that assess aspects related to the recovery approach. A total of 95 measures were found, a large proportion of which were not targeted for carers of those with psychosis or schizophrenia. Of the 10 measures considered relevant for this review, half were developed specifically for use with carers of those with psychosis or schizophrenia, 30% were developed for carers of those with a serious mental illness and 20% were developed for carers of those with a serious mental illness and either dementia or a physical impairment.

### Recommendations for Instrument Selection

Out of the 10 measures, the CarerQol was the most frequently evaluated with three studies assessing its validity. However, these studies only assessed content validity and hypothesis testing and therefore did not score highly on the COSMIN checklist. Instead, the three measures that scored highly on the COSMIN checklist and thus showed the strongest psychometric properties were the CWS, the ECI, and the SCQ. The CWS was found to have excellent internal consistency, content validity and structural validity, with good hypothesis testing and a fair level of reliability. The ECI showed excellent internal consistency, content validity and structural validity, and good hypothesis testing. The SCQ demonstrated excellent internal consistency, content validity, structural validity, a good level of reliability and fair hypothesis testing. It should be noted that the COSMIN results only provide limited guidelines on instrument selection. There are two other important factors when considering instrument selection for this review, public involvement in the questionnaire design, and how well it assesses elements of personal recovery for carers.

Public involvement in the development of a measure, directly relates to the relevance and content validity of the measure (Slevin et al., 1988; Testart et al., 2013; Zendjidjian and Boyer, 2014). It is seen as good practice and crucial to current measure development processes (Sklar et al., 2013), as it adds to the robustness of the research and is recommended by policy and funding directives (Shippee et al., 2015). Public involvement in the development of the 10 measures was mixed: five showed “good” to “excellent” public involvement with only three demonstrating “excellent” public involvement by involving carers at every stage of the development process. The latter aligns with the recommendations made by Rat et al. (2007) who argue that it provides the most valid set of items for respondents. The remaining five measures showed either poor or no public involvement at any stage of the measure development. A similar comprehensive review of outcome measures for carers by Harvey et al. (2008) also found that a relatively low proportion of measures (8 out of 25) were developed with public involvement. Harvey et al. (2008) did note a greater level of public involvement in the more recently developed measures and it is clearly seen as good practice in measure development (Streiner et al., 2015). However, this was not echoed in the present review as some of the most recent measures like the Family Mental Health Recovery Tool developed in 2016 showed a limited amount of public involvement in the development process, and the measure that demonstrated one of the best levels of public involvement, the ECI, was developed in 1996.

The second important factor when considering instrument selection for this review is how well each measure fits within the recovery framework. The Family Mental Health Recovery Tool is the only measure that has a good fit with the recovery framework, however, it is not currently available for use outside of the “Families Healing Together” intervention (Rue et al., 2016). The CareQol, ECI and FLLISS all have a substantial focus on the burden and stress of caregiving and are therefore not considered useful in assessing recovery. Even though the ECI is one of the most comprehensively validated measures and scores highly on the COSMIN checklist, it only partially fits the recovery framework assessing only two positive aspects of caring; rewarding personal experiences, and good aspects of the relationship with the person being cared for. The CWS incorporates several aspects related to personal recovery in the carer wellbeing subscale such as: day to day coping, interpersonal relationships, physical and emotional wellbeing, and feelings of personal safety. The SCQ also provides a comprehensive set of items that assesses aspects relating to recovery such as: the “humanistic impact” on the social, emotional, and daily life of life of the carer, and the aspects and perceptions related to the caregiver role. Our recommendation of the best measures to use to assess personal recovery would be either the CWS or SCQ or a combination of the two as they show strong psychometric properties, cover a range of relevant aspects related to personal recovery, and demonstrated a good level of public involvement in the development of the questionnaires. However, using multiple measures to assess personal recovery still does not assess the multi-dimensional nature of the recovery concept, and it could become burdensome for carers to complete. A solution to this would be the development of a new outcome measure with a specific focus on recovery for carers that could be used in future research studies as a more appropriate way to assess this construct.

### Strengths and Limitations

The COSMIN has several strengths as a robust and rigorous assessment tool that was developed by an international team of experts (Mokkink et al., 2010). It is becoming recognized as the “gold standard” and is a popular tool for many health-related systematic reviews (Rosenkoetter and Tate, 2018). Thus, this review has used the strongest quality appraisal possible. This review is also strengthened by the fact that it goes beyond reporting on the COSMIN findings, by assessing another important aspect of good practice in questionnaire design, public involvement in research.

This review presented a challenge in trying to apply the concept of personal recovery to a carer population, which has been both a strength and limitation. Because of the complex nature of how to define personal recovery, the research team devised a way to operationalize the concept by reviewing the definitions of recovery as outlined by the key authors in this area: Anthony (1993), Resnick et al. (2005), Slade (2009) the CHIME framework outlined by Leamy et al. (2011). The key concepts and linguistic terms were then incorporated into a checklist (see Supplementary Material) and formed the basis of the search terms of this review. This can be seen as a strength as it provides a transparent overview of our understanding of the key features of recovery for carers.

However, by focusing on elements of recovery we may have been overly inclusive in terms of papers identified as being potentially relevant. Note that 95 measures were identified initially, but only ten of these could be related directly to recovery in some way. This may raise questions about the focus of our search strategy. In the searches, the terms used to describe the target population brought back results for carers from different clinical populations (physical and mental health). Two searches were used with the Boolean operator “AND,” however, this still brought back irrelevant studies for this review. On a positive note, this means that it is unlikely that any relevant studies were missed.

A limitation of this review is a potential selection bias due to the choice to only include English language measures due to lack of funding to employ translators. This review also excluded translated versions of measures originally developed in English, and measures that were developed in a foreign language, as there appeared to be many non-English language measures that this would warrant a separate review. However, there were two potentially relevant measures that were excluded because they were developed and validated in a non-English language sample. The Scale for Positive Aspects of Caregiving Experience (SPACE) (Kate et al., 2012) was validated in Hindi, and the Schizophrenia Caregiver Quality of Life Questionnaire (S-CGQol) (Richieri et al., 2011) was validated in French. This review did not include short form measures either as it was felt that the reduced number of items would affect the content validity of the measure and considering that measures only partly assess aspects of recovery this would prove to be problematic. A further limitation of this review was that it was not possible for the second reviewer to carry out the full COSMIN assessment on all papers due to time constraints, however, the second reviewer carried out a 20% check of the work with a good level of agreement to the first author.

## Conclusion

This review set out to identify all self-report measures that have been developed for use with carers of those with psychosis or schizophrenia and that assess aspects of personal recovery. It seems that in fact, there may be no measure targeting carers' recovery per se, despite its potential importance. The authors therefore set out to examine carer measures that to some extent measure specified aspects of “carer recovery” and attempt to encapsulate this issue across available instruments. A small number of measures are available that combined, could be used to assess personal recovery for carers. The only measure specifically developed to assess recovery, the Family Mental Health Recovery Evaluation Tool is not currently available to clinicians or researchers. To get the most comprehensive assessment of recovery using the measures that are currently available would mean that a selection of measures would need to be used together which would be time consuming and burdensome for respondents to complete. For example, if the CWS, the ECI, SCQ, and the SNQ were to be used as a set of questionnaires to assess recovery, this would involve the participants completing an approximate total of 160 items. One solution would be to combine selected subscales from each of the various measures to form a new measure, however, this would still need to be validated as a separate measure and would still not cover all the aspects related to the concept of personal recovery. This review highlights the need for further research in this area, and the potential development of a new measure that is specifically focused on assessing personal recovery for carers especially considering the recent call for more support for carers on their “parallel” recovery journey (Wyder and Bland, 2014; Lovelock, 2016; Poon et al., 2017). The COSMIN checklist provided a useful quality assessment for this review despite some failings. It enabled an overall quality assessment of the psychometric properties of each outcome measure to be assessed. It is also clear that public involvement is important at every stage in the development of a measure if this is to provide a tool that is valid and relevant for the target population.

## Data Availability Statement

The original contributions presented in the study are included in the article/Supplementary Material, further inquiries can be directed to the corresponding author/s.

## Author Contributions

CH designed the study, wrote the protocol, carried out the literature searches, screened the articles, carried out the data extraction, completed the COSMIN checklist analysis, and wrote the manuscript. WS and SJ provided input into the study design and protocol, contributed to, and approved the final manuscript. NA screened articles, contributed to the analysis, contributed to, and approved the final manuscript. KP contributed to and approved the final manuscript. All authors contributed to the article and approved the submitted version.

## Funding

This systematic review has been completed as part of a PhD studentship, funded by the Economic and Social Research Council (ESRC). The funder had no input into the study design, collection, analysis or interpretation of the data, writing of the manuscript or decision as to where to submit the manuscript for publication.

## Conflict of Interest

The authors declare that the research was conducted in the absence of any commercial or financial relationships that could be construed as a potential conflict of interest.

## Publisher's Note

All claims expressed in this article are solely those of the authors and do not necessarily represent those of their affiliated organizations, or those of the publisher, the editors and the reviewers. Any product that may be evaluated in this article, or claim that may be made by its manufacturer, is not guaranteed or endorsed by the publisher.
