# Supplementary material for: Self-Report Measures Assessing Aspects of Personal Recovery in Relatives and Other Informal Carers of Those With Psychosis: A Systematic Review
Source: Front Psychol. 2022 Jul 15;13:926981. doi: 10.3389/fpsyg.2022.926981 (PMC9335122; doi:10.3389/fpsyg.2022.926981)
Supplement: Supplementary file 1 [file Data_Sheet_1.docx]

**Psychosis Carers Recovery Measures Review**

**Appendices and Supplementary material**

## Appendix A - Inclusion and exclusion criteria for title and abstract screening

| Criteria | Inclusion | Exclusion |
| --- | --- | --- |
| Duplicate | Not a duplicate | A duplicate |
| Abstract/  Language | Abstract and paper title provided in the English Language.  Paper appears to have been published in English language, in an English language journal. | Abstract or title either not in English, or non-existent.  Paper appears to be published in a language other than English. |
| Publication type | Primary research studies, measure validation papers, measure development papers, systematic review, meta-analysis, conference proceedings, grey literature, peer reviewed papers.  Papers using a quantitative approach or mixed methods as their primary methodology. | Opinion/discussion piece, book review, a noting of a correction to a study, study protocol, unpublished dissertations, and theses.  Papers using a qualitative approach as their primary research methodology. |
| Date of publication | Any | None |
| Population | Adult carers/relatives/friends – may include: parents, spouses, partners, grandparents, siblings, extended family, close friends in a caring role. | Paid carers, in-patient care staff, young carers, relatives under the age of 18 years old. Young carers (below age 18) were excluded as they have a different and more complex care experience to adult carers that may include more input from external agencies. |
| Clinical group (service user) | The service user of the relative/carer must have a diagnosis of a psychosis related serious mental health problem and must be an adult over the age of 16 years.  Includes:  Schizophrenia (all types), acute and chronic psychosis, first episode psychosis, psychotic episodes.  Psychotic features of other serious mental illness, such as bipolar disorder and personality disorder, where this is the main focus of the paper. | All forms of dementia. Any form of learning disability. Any form of developmental disorder such as: language disorders, learning disorders, motor disorders, autistic spectrum disorders and ADHD. Any physical health problems such as cancer, stroke, head injury etc.  Those under the age of 16 years. |
| Outcome measures | Any formal set of questions that have been designed and tested for use with relatives and carers.  Includes self-report measures such as: questionnaires, surveys, outcome assessments, instruments and rating scales.  Also includes measures completed by a health professional through verbal questioning of the relative, such as in a structured interview. | Measures designed for populations other than relatives, even if those measures are commonly used in research studies with relatives, for example: The General Health Questionnaire (GHQ).  Any measures assessing the service user.  Measures that include a section with open ended questions or semi-structured interviews.  Measures developed or translated into another language. It will be assumed that measures that have been used in foreign language research studies will have been translated into a foreign language, unless it is stipulated in the methods sections that English language measures were used. |
| Concepts being assessed in the outcome measures | Relatives’ own personal recovery as relates to the ‘recovery approach’ and ‘mental health recovery’.  Aspects of recovery such as:  Hope, optimism, goals, relationships, identity, meaning, personal responsibility, ‘full engagement with life’, empowerment, knowledge, ‘life satisfaction’, self-direction, ‘full potential’, person-driven, ‘peer support’, ‘support groups’, community, strengths, respect, ‘motivation to change’, ‘positive thinking’, ‘valuing success’, aspirations, ‘positive sense of identity’, ‘quality of life’, ‘meaningful life’, ‘meaningful social roles’, ‘rebuilding life’, employment, self-efficacy, coping, adaptability  Other aspects relating to the positive aspects of caregiving: social support, interpersonal support, family satisfaction, family adaptability and cohesion, spirituality and personal growth. | Physical health, general health, carer burden, family burden, negative aspects of caregiving, caregiving hassles, stress scales, strain scales, caregiver distress, depression, anxiety, personality inventories, medical outcomes. |

## Appendix B – Example search strategy

Search strategy: PsychINFO (EBSCOHost)

1. [POPULATION] (Using thesaurus subject terms) DE "Caregivers” OR DE "Family" OR DE "Extended Family" OR DE "Family Members" OR DE "Adult Offspring" OR DE "Biological Family" OR DE "Daughters" OR DE "Sons" OR DE "Parents" OR DE "Fathers" OR DE "Mothers" OR DE "Siblings" OR DE "Brothers" OR DE "Sisters" OR DE "Spouses" OR DE "Husbands" OR DE "Wives" OR DE "Significant Others"
2. [POPULATION] (Using key words) carer* OR relative* OR families OR 'family caregiver*'
3. Thesaurus subject terms OR key words
4. [POPULATION] (Using thesaurus subject terms) DE "Psychosis" OR DE "Acute Psychosis" OR DE "Affective Psychosis" OR DE "Chronic Psychosis" OR DE "Postpartum Psychosis" OR DE "Reactive Psychosis" OR DE "Schizophrenia" OR DE "Acute Schizophrenia" OR DE "Paranoid Schizophrenia" OR DE "Mental Disorders" OR DE "Bipolar Disorder" OR DE "Schizoaffective Disorder" OR DE "Chronic Mental Illness" OR DE "Personality Disorders"
5. [POPULATION] (Using key words) psychosis OR psychoses OR psychotic OR 'psychotic disorder' OR schizophren*
6. Thesaurus subject terms OR key words
7. [TYPE OF INSTRUMENT] (using thesaurus subject terms) DE "Measurement" OR DE "Psychological Assessment" OR DE "Behavioral Assessment" OR DE "Cognitive Assessment" OR DE "Emotional Assessment" OR DE "Motivation Measures" OR DE "Stress and Coping Measures" OR DE "Questionnaires" OR DE "Surveys" OR DE "Data Collection"
8. [TYPE OF INSTRUMENT] (using key words) "outcome measure*" OR "instrument* and assessment*" OR "measurement scale*" OR "rating scale*" OR "survey*" OR "questionnaire*" OR "patient reported outcome measure" OR "patient reported outcome" OR "self-report measure"
9. Thesaurus subject terms OR key words
10. [CONSTRUCT] (key words only) recovery OR "recovery in mental health" OR "recovery model mental health" OR "mental health recovery" OR hope OR optimism OR meaning OR purpose OR empowerment OR "life satisfaction" OR "positive thinking" OR "valuing success" OR aspirations OR "positive sense of identity" OR "quality of life" OR "meaningful life" OR "rebuilding life" OR self-efficacy OR coping OR adaptability OR adjustment
11. Final Search using searches 3 AND 6 AND 9 AND 10
12. Limit to Age 18+ (Adulthood), English Language, Human Participants

**Appendix C - Table 5 Quality criteria for good measurement properties modified from Terwee et al. (2007) and DeVet et al. (2011)**

| Measurement property | Rating* | Criteriaª |
| --- | --- | --- |
| Content validity  (including face validity) | +  ?  - | All items refer to relevant aspects of the construct to be measured AND are relevant for the target population AND are relevant for the purpose of the measurement instrument AND together comprehensively reflect the construct to be measured  Not all information for ‘+’ reported  Criteria for ‘+’ not met |
| Structural validity | +  ?  - | *Factors should explain at least 50% of the variance or adequate or good fit by goodness-of-fit criteria for a confirmatory factor analysis (CFA) or exploratory factor analysis (EFA).*  Not all information for ‘+’ reported  Criteria for ‘+’ not met |
| Internal consistency | +  ?  - | At least limited evidence for unidimensionality or positive structural validity AND Cronbach’s alpha(s) ≥0.70 and ≤0.95  Not all information for ‘+’ reported OR conflicting evidence for unidimensionality or structural validity OR evidence for lack of unidimensionality or negative structural validity  Criteria for ‘+’ not met |
| Reliability | +  ?  - | ICC or weighted Kappa ≥0.70  ICC or weighted Kappa not reported  Criteria for ‘+’ not met |
| Construct validity  *(Hypothesis testing)* | +  ?  - | *Convergent or divergent validity tested AND good correlations reported*  No correlations with instrument(s) measuring related construct(s) AND no differences between relevant groups reported  Criteria for ‘+’ not met |

**Supplementary material 1: Recovery Terms Checklist**

One aim of the review was to include those measures that had at least some potential focus on personal recovery. This raised the question as to how this factor should be appraised when selecting papers. To this end, a checklist was developed which aimed to cover items or subscales pertaining to personal recovery. Looking at definitions of recovery and recovery outcomes allowed the development of such a checklist. Sources for this checklist included: Anthony’s (1993) definition, the CHIME framework outlined by Leamy et al. (2011) and the descriptions by Resnick et al. (2005); Slade (2009). (See the main paper for the full references).

Key terminology was extracted from the texts outlined above. These were then grouped by the lead author into categories. All of the terminology identified formed the basis of the search strategy and inclusion and exclusion criteria for this review.

**Checklist for Personal Recovery concepts**

Outcome measures will need to address aspects related to personal recovery as it relates to the ‘recovery approach’ and ‘mental health recovery’

| Category | Aspects/key terms |
| --- | --- |
| Hope | Relating to ideas of optimism, ‘positive thinking’, valuing success, full potential, aspirations |
| Goals | Goals for the future, self-direction, full potential, person-driven, motivation to change, rebuilding life, aspiration, full potential, employment |
| Relationships | Relating to family relationships, social networks, meaningful social roles, family adaptability, family cohesion, respect |
| Support | Peer support, peer support groups, community support, social support, interpersonal support, employment |
| Meaning | Spirituality, meaningful life, meaningful social roles, personal growth, quality of life |
| Identity | Change in identity, understanding oneself, personal responsibility, empowerment, self-aware, self-direction, person driven, positive sense of identity, self-efficacy |
| Adaptation | Coping, adaptability, rebuilding life, quality of life, strength, gaining knowledge, empowerment, life satisfaction |
